# Comparison of gene expression response to neutron and x-ray irradiation using mouse blood

**DOI:** 10.1186/s12864-016-3436-1

**Published:** 2017-01-03

**Authors:** Constantinos G. Broustas, Yanping Xu, Andrew D. Harken, Guy Garty, Sally A. Amundson

**Affiliations:** 1 Center for Radiological Research, Columbia University Medical Center, 630 West 168th Street, New York, NY 10032 USA; 2 Radiological Research Accelerator Facility, Columbia University, Irvington, NY 10533 USA

**Keywords:** Gene expression, Microarrays, Neutron, x-ray, Radiation biodosimetry

## Abstract

**Background:**

In the event of an improvised nuclear device detonation, the prompt radiation exposure would consist of photons plus a neutron component that would contribute to the total dose. As neutrons cause more complex and difficult to repair damage to cells that would result in a more severe health burden to affected individuals, it is paramount to be able to estimate the contribution of neutrons to an estimated dose, to provide information for those making treatment decisions.

**Results:**

Mice exposed to either 0.25 or 1 Gy of neutron or 1 or 4 Gy x-ray radiation were sacrificed at 1 or 7 days after exposure. Whole genome microarray analysis identified 7285 and 5045 differentially expressed genes in the blood of mice exposed to neutron or x-ray radiation, respectively. Neutron exposure resulted in mostly downregulated genes, whereas x-rays showed both down- and up-regulated genes. A total of 34 differentially expressed genes were regulated in response to all ≥1 Gy exposures at both times. Of these, 25 genes were consistently downregulated at days 1 and 7, whereas 9 genes, including the transcription factor E2f2, showed bi-directional regulation; being downregulated at day 1, while upregulated at day 7. Gene ontology analysis revealed that genes involved in nucleic acid metabolism processes were persistently downregulated in neutron irradiated mice, whereas genes involved in lipid metabolism were upregulated in x-ray irradiated animals. Most biological processes significantly enriched at both timepoints were consistently represented by either under- or over-expressed genes. In contrast, cell cycle processes were significant among down-regulated genes at day 1, but among up-regulated genes at day 7 after exposure to either neutron or x-rays. Cell cycle genes downregulated at day 1 were mostly distinct from the cell cycle genes upregulated at day 7. However, five cell cycle genes, *Fzr1*, *Ube2c*, *Ccna2*, *Nusap1*, and *Cdc25b,* were both downregulated at day 1 and upregulated at day 7.

**Conclusions:**

We describe, for the first time, the gene expression profile of mouse blood cells following exposure to neutrons. We have found that neutron radiation results in both distinct and common gene expression patterns compared with x-ray radiation.

**Electronic supplementary material:**

The online version of this article (doi:10.1186/s12864-016-3436-1) contains supplementary material, which is available to authorized users.

## Background

The goal of radiation biodosimetry is to accurately predict radiation dose, as a surrogate of radiological injury, in a large-scale radiation emergency. Most approaches focus on the first week after an event, when radiological triage is most needed. A wide range of different methods have been used to identify cellular responses to radiation, ranging from cytogenetic measurements, specifically dicentric assays, to high throughput proteomic, metabolomic, and transcriptomic profiling [1]. Several groups have reported gene expression-based analyses, with the development of predictive gene sets that correlate with dose [2–5].

Radiation quality plays an important role in driving gene expression, and different signaling pathways may be triggered in response to the different types of irradiation. In addition, various genes and biological functions are induced in cells at different time points after radiation exposure [6–8]. To date, most of the gene expression signatures for the purpose of radiation biodosimetry have been obtained in response to photon (γ- and x-rays) exposure. However, there have been a limited number of studies that have compared gene expression profiles in mice or human blood cells after exposures involving heavy ions and α-particles, the latter representing isotopes likely to be used in a radiological dispersal device [9]. For example, using human peripheral blood mononuclear cells, Chauhan et al. [10] found that the gene expression profile induced by α-particle radiation was very similar to the x-ray responses, despite the fact that α-particles are characterized by a higher linear energy transfer coefficient compared with x-rays [11]. In contrast, comparison of α-particle and x-ray irradiation impact on human tumor and endothelial cells [12], as well as human epidermal keratinocytes [13] produced distinct gene expression profiles.

An improvised nuclear device (IND) detonation is capable of producing a mixture of γ-rays and fast neutrons. Furthermore, as neutrons generally have a higher Relative Biological Effectiveness (RBE) for most physiological endpoints, it is important to understand the impact that neutrons would have on the biodosimetry methods that are being developed for medical triage purposes. Radiation affects multiple biological processes, including immune system and inflammatory responses, cell cycle progression, cell death, DNA repair, as well as metabolism. However, the impact of neutron radiation on any of these processes has not been studied in detail. This study presents an initial characterization of gene expression responses following exposure to IND-spectrum neutrons or x-rays.

In the present study, gene expression signatures were analyzed after in vivo irradiation of mice with either neutrons or x-rays. The doses used were selected based on the limited RBE information available for IND-spectrum neutrons. Using the in vitro cytokinesis-block micronucleus assay [14] in peripheral human blood lymphocytes, Xu et al. [15] calculated the RBE of our neutron spectrum to be between 3 and 5 for micronucleus induction with 250 kVp x-rays used as the reference radiation, with similar results for micronucleus induction in mice (Helen Turner, personal communication). We therefore exposed mice to 0.25 Gy or 1 Gy of neutron radiation and, separately, 1 Gy or 4 Gy of x-ray radiation. We describe biological processes significantly over-represented only in the neutron response, or only in the x-ray radiation response, as well as processes shared by both types of radiation. The ultimate goal is to investigate whether we can separately estimate the photon and the neutron component after a mixed photon/neutron exposure, and to develop gene expression signatures capable of discriminating between neutron and photon exposures.

## Methods

### Animals and irradiation

All animal experiments were conducted in accordance with applicable federal and state guidelines and were approved by the Columbia University Institutional Animal Care and Use Committee (IACUC) (approval number AC-AAAG4356). Male C57BL/6 mice were received from Charles River Laboratories (Frederick, MD) and quarantined for 2 weeks before irradiation at 8-10 weeks of age. Twelve mice were used for each treatment, with six being sacrificed 1 day after treatment, and the other six being sacrificed 7 days after treatment. Samples were lost from three mice (one each of the day 1 controls, 1 Gy x-ray at day 7, and 4 Gy x-ray at day 1) due to clotting during blood draw.

Mice were either sham-irradiated or exposed to 0.25 Gy or 1 Gy neutron beam or to 1 Gy or 4 Gy x-rays. Neutron irradiations were performed using the accelerator-based IND-spectrum neutron source [15] at the Radiological Research Accelerator Facility (RARAF). Briefly, a mixed beam of atomic and molecular ions of hydrogen and deuterium was accelerated to 5 MeV and used to bombard a thick beryllium target. The neutrons emitted at 60° to the ion beam axis have a spectrum that closely mimics the Hiroshima spectrum at 1.5 km from the epicenter [16].

For irradiation, six to eighteen mice were placed in holders in adjacent positions on an eighteen position Ferris wheel, rotating around the beryllium target at a distance of 17.5 cm, at an angle of 60° to the particle beam. The mouse holders, based on 50 ml conical tubes, are designed to maintain a horizontal orientation as the wheel rotates, providing an isotropic irradiation, while maintaining the mice in an upright orientation. With a wheel rotation speed of about 2 min per revolution, the dose rate was adjusted so that the smallest dose was delivered in 5 rotations (approximately 10 min). The mouse holders were reversed end-to-end midway through exposure, to correct for any angular variation in dose rate so that the front and back of the mouse receive equivalent doses. When fewer than 18 mice were on the wheel, two 50 ml tubes containing Lucite phantoms were placed at either end of the string of occupied mouse holders in order to ensure a uniform scatter dose. Irradiations were performed with a total beam current of 18 μA on the target, resulting in a dose rate of 1.55 Gy/h of neutrons and 0.4 Gy/h of γ rays. In order to allow the mice to acclimate to the restraining conditions of the holders and reduce stress during treatment, the mice were placed in the holders in two separate sessions at 3 days and 1 day prior to irradiation. On the second training session one day prior to the actual irradiation, the mice in the holders were placed on the irradiation wheel. Sham-irradiated control mice underwent the same holder acclimation protocol, and on the day of exposure they were placed in the holders on the wheel with the beam off for mock treatment. Controls were performed prior to neutron irradiation, to eliminate dose from activation of materials at the endstation.

For x-ray irradiation, mice were exposed to 1 Gy or 4 Gy of x-rays from a Westinghouse Coronado orthovoltage x-ray machine operating at 250 kVp and 15mA with a 0.5mm Cu + 1mm Al filter. The dose rate at the mouse location was 1.23 Gy/min, as determined using a Victoreen model 570 condenser R meter with a 250r chamber. After dose administration, mice were housed in micro-isolator cages until the time of sacrifice and blood draw.

No significant changes in cell death or constitution of white blood cells were observed after irradiation.

### Blood Collection and RNA isolation

Blood was collected by cardiac puncture at the time of euthanasia at days 1 and 7 post-irradiation. Each sample (~0.4 ml blood) was added to a 15 ml centrifuge tube that contained 1.6 ml of PAXgene Blood RNA stabilization and lysis solution (PreAnalytix GmBH, catalog # 762165) and mixed thoroughly, while a small amount of blood was added to anti-coagulant containing tubes for blood count using a Genesis hematology system (Oxford Science). After collection was complete, blood was mixed gently but thoroughly and the tubes were incubated at 4 °C for 24 h. Blood samples were allowed to reach room temperature for 2 h before proceeding to RNA isolation. RNA was purified following the PAXgene RNA kit recommendations with on-column DNase I treatment. Globin RNA was reduced using the Ambion GLOBINclear-mouse/rat kit (Thermofisher). RNA yields were quantified using the NanoDrop ND1000 spectrophotometer (Thermofisher) and RNA quality was checked by the 2100 Bioanalyzer (Agilent). High quality RNA with an RNA integrity number of at least 7.0 was used for microarray hybridization.

### Microarray hybridization and data analysis

Cyanine-3 labeled cRNA was prepared using the One-Color Low input Quick Amp Labeling kit (Agilent). Dye incorporation and cRNA yield were measured with a NanoDrop ND1000 spectrophotometer (Thermofisher). Labeled cRNA was fragmented and hybridized to Agilent Mouse Gene Expression 4x44K v2 Microarray Kit (G4846A). Slides were scanned with the Agilent DNA microarray scanner (G2505B) and the images were analyzed with Feature Extraction software (Agilent) using default parameters for background correction and flagging non-uniform features.

Background-corrected hybridization intensities were imported into BRB-ArrayTools, version 4.5.0, log_2_-transformed and median normalized. Non-uniform outliers or features not significantly above background intensity in 25% or more of the hybridizations were filtered out. In addition, a minimum 1.5-fold change in at least 20% of the hybridizations was set as a requirement. Furthermore, probes were averaged to one probe per gene and duplicate features were reduced by selecting the one with maximum signal intensity. A final set of 16,489 features was used in subsequent analyses. The microarray data is available through the Gene Expression Omnibus with accession number GSE85323.

Class comparison was conducted in BRB-ArrayTools to identify genes differentially expressed (*p* < 0.001) between radiation exposed samples and time-matched unirradiated controls using a random variance *t*-test [17]. Time-matched controls were also compared with each other but no significantly differentially expressed genes (FDR > 0.05) were found. Genes with *p*-values less than 0.001 were considered statistically significant. The false discovery rate (FDR) was estimated for each gene by the method of Benjamini and Hochberg [18], to control for false positives. All genes used in this analysis had a FDR of less than 0.05.

Hierarchical clustering of microarray gene expression data was performed with the Dynamic Heatmap Viewer of the BRB-ArrayTools software using a one minus correlation metric and average linkage. Genes differentially expressed following exposure to 1 Gy neutron, and 1 and 4 Gy x-ray at either day 1 or day 7 post-irradiation (a total of 494 probesets) were used to construct the heatmap.

### Gene ontology analysis

Lists of genes that were either significantly overexpressed or underexpressed compared with controls were imported separately into the Database for Annotation, Visualization, and Integrated Discovery (DAVID), version 6.7 [19], to identify enriched biological processes and gene ontology (GO) terms using the functional annotation tool. Benjamini corrected *p* values of < 0.05 were considered significant. To construct Tables 2, 3, and 4, redundant GO terms were grouped using the REVIGO software [20].

### Quantitative RT-PCR

cDNA was prepared from total globin-cleared RNA using the High-Capacity cDNA Archive kit (Thermofisher). Quantitative real-time PCR (qRT-PCR) was performed for five genes (*Ube2c*, *Fzr1*, *Ccna2*, *Cdc25b*, and *Nusap1)* using Taqman assays (Thermofisher). *Fzr1* (Mm00517239_m1), *Ccna2* (Mm00438063_m1), *Cdc25b* (Mm00499136_m1), *Nusap1* (Mm00505601_m1) were pre-designed validated assays. Mouse *Ube2c* primers were designed using the PrimerQuest Tool (Integrated DNA Technologies), and the sequences were as follows: forward primer, CTGCTAGGAGAACCCAACATC; reverse primer: GCTGGAGACCTGCTTTGAATA; and probe: CTTTGAACACACACGCTGCGGAAC. A β-actin assay (Mm00607939_s1) was also performed alongside as control. The gene expression validation experiments were conducted with 20 ng cDNA using Universal PCR Master Mix (Thermofisher) in an ABI 7900 Real Time PCR system. After an initial activation at 50 °C for 2 min and 95 °C for 10 min, the PCR reaction was performed by 40 cycles of 95 °C for 15 s and 60 °C for 60 s. Relative fold-induction was calculated by the 2^-ΔΔCT^ method [21], using SDS version 2.3 (Thermofisher). Data were normalized to *β-actin* gene expression levels.

## Results

### Microarray experiments

Mice were either sham-irradiated or exposed to 0.25 Gy or 1 Gy of neutron irradiation or 1 Gy or 4 Gy of x-ray irradiation.. All animals remained in apparent good health, with no adverse events noted during the course of the study. Total blood counts were within the normal range in controls and all animals prior to irradiation and at days 1 and 7 post irradiation.

Global gene expression was measured in the blood of mice sacrificed 1 and 7 days post-irradiation using Agilent Whole Mouse Genome Microarrays. Class comparison using BRB-ArrayTools identified a total of 7285 and 5045 genes differentially expressed (*p* < 0.001, false discovery rate (FDR) < 5%) between unirradiated controls and neutron or x-ray exposed samples, respectively (Additional file 1). The number of differentially expressed genes varied with time, dose, and radiation quality (Table 1). Following neutron exposures, genes were predominantly down-regulated, with nearly all differentially expressed genes being under expressed on day 7 post-exposure. Up- and down-regulated genes were more evenly divided after x-ray exposure.

**Table 1:** Differentially expressed genes

Neutron				X-rays			
Sample	Up	Down	Total	Sample	Up	Down	Total
N025d1	15	31	46	X1d1	378	387	765
	33%	67%			49.50%	50.50%	
N025d7	17	674	691	X1d7	167	396	563
	2.50%	97.50%			30%	70%	
N1d1	885	1071	1956	X4d1	1389	2188	3577
	45%	55%			39%	61%	
N1d7	614	5612	6226	X4d7	1094	491	1585
	10%	90%			69%	31%	

Significantly differentially expressed genes in mouse blood after neutron or x-ray treatment relative to unirradiated controls (*p* < 0.001). Percent of upregulated and downregulated genes are shown. N025d1: 0.25 Gy neutron, day 1; N025d7: 0.25 Gy neutron, day 7; N1d1: 1 Gy neutron, day 1; N1d7: 1 Gy neutron, day 7; X1d1: 1 Gy X-rays, day 1; X1d7: 1 Gy X-rays, day 7; X4d1: 4 Gy X-rays, day 1; X4d7: 4 Gy X-rays, day 7

**Fig. 1:**
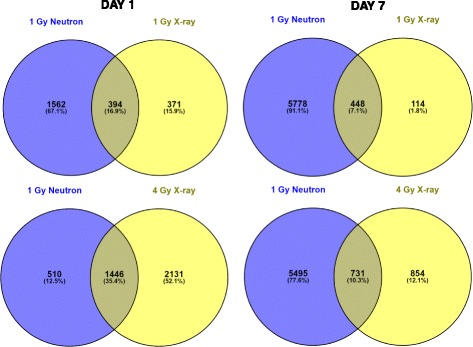
Differentially expressed genes. Venn diagram showing overlap patterns of genes that are differentially expressed at days 1 and 7 in response to 1 Gy neutron vs. 1 or 4 Gy x-ray irradiation

**Fig. 2:**
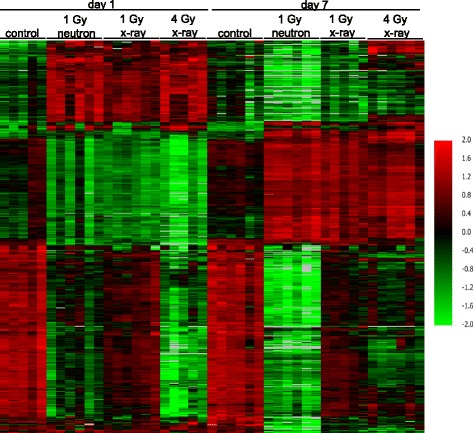
Heat map illustrating relative gene expression at days 1 and 7 post-irradiation. Hierarchical cluster analysis was performed on 494 probesets responding to both 1 Gy neutrons and 1 and 4 Gy x-rays relative to unexposed controls at either 1 or 7 days after exposure. *Red* indicates high expression, *green* indicates low expression as indicated in the color key. Each row represents one gene and each column represents an individual mouse, ordered by exposure dose and radiation type as labeled in the figure

We also sought to determine the extent to which neutrons and x-rays share common differentially expressed genes. Venn diagrams revealed the overlap of differentially expressed genes after 1 Gy neutron or x-ray irradiation to be, for neutron, 20.1% and 7.8% at days 1 and 7, respectively, and, for x-rays, 51.5% and 79.7% at days 1 and 7, respectively. Comparing the overlap of differentially expressed genes between 1 Gy neutron and 4 Gy x-rays, we found the percentages to be 73.9%, 11.7% (neutron) and 40.4%, 46.1% (x-rays) at days 1 and 7, respectively (Fig. 1). In all, 272 genes were common to the response to 1 Gy neutrons and both 1 and 4 Gy x-rays at day 1 after exposure, and 256 were common to all three conditions at day 7 after exposure. Hierarchical clustering of these most consistently responsive genes was visualized as a heat map to compare the relative expression across samples (Fig. 2). Patterns of differential expression were seen to vary by radiation quality and dose, and by time since exposure, showing general consistency between replicates. Control samples showed no obvious differences in expression between days 1 and 7.

**Fig. 3:**
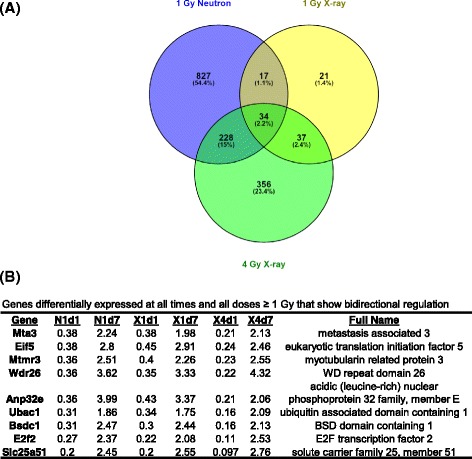
Genes differentially expressed at both times. **a**, Venn diagram showing the number of genes differentially expressed at both time points after exposure to 1 Gy neutrons or 1 or 4 Gy x-rays. **b**, list of bi-directionally regulated genes common to all conditions other than 0.25 Gy neutron (*P* < 0.001) with fold change relative to controls for each dose and time. N1d1: 1 Gy neutron, day 1; N1d7: 1 Gy neutron, day 7; X1d1: 1 Gy x-rays, day 1; X1d7: 1 Gy x-rays, day 7; X4d1: 4 Gy x-rays, day 1; X4d7: 4 Gy x-rays, day 7

Finally, we searched for genes with sustained differential expression at both day 1 and day 7. The higher doses produced the most sustained responses, with 1105 genes differentially expressed at both times after 1 Gy neutron and 555 after 4 Gy x-ray exposure. Only 2 genes were regulated at both times after 0.25 Gy neutrons, and 110 after 1 Gy x-ray exposure. Thirty-four genes were common to both times after 1 Gy neutron and 1 and 4 Gy x-ray exposure (Fig. 3a). Of these genes, 25 were uniformly downregulated. Interestingly, the remaining nine genes showed a bi-directional temporal response to irradiation. These genes were downregulated at day 1 post-irradiation, whereas they were upregulated at day 7 (Fig. 3b). One of the genes temporally regulated was *E2f2* (Fig. 3b), a member of the E2F family of transcription factors that play important roles in the control of the cell cycle. We also performed a search for other differentially expressed E2f factors and found that, unlike *E2f2*, *E2f1*, *E2f3*, and *E2f4* were downregulated only after 1 Gy neutron exposure at day 7, showing respectively a 0.44-, 0.18-, and 0.38-fold change from control levels. In contrast, *E2f1* and *E2f8* showed up regulation (2.38- and 2.05-fold) after 4 Gy x-rays at day 7.

### Gene ontology analysis

Differentially expressed genes were functionally classified into gene ontology categories by the Database for Annotation, Visualization, and Integrated Discovery (DAVID) [19] analysis. Biological processes over-represented among differentially expressed genes with a Benjamini-corrected *p*-value of less than 0.05 were considered significant (Additional file 2). Radiation exposure to the lower dose of neutrons (0.25 Gy) produced few biological processes, and these were primarily related to immune system response. Therefore, we focused our analysis on the response to the higher dose exposures (1 Gy neutron and 1 Gy and 4 Gy x-rays). The top biological processes among differentially expressed genes were related to immune system response, which was significantly enriched among genes downregulated in response to both 4 Gy of x-rays and 1 Gy neutron irradiation after 1 and 7 days, and after 1 Gy x-rays at day 1.

A significant class of biological processes among downregulated genes responding to both x-rays (Table 2) and, particularly, neutrons (Table 3) was the one related to DNA and RNA metabolism and processing. In the x-ray data, biological processes such as DNA replication and regulation of transcription were significant only among genes downregulated on day 1 after 4 Gy of x-rays. The profile of DNA/RNA-related biological processes significant at day 1 after 1 Gy neutron irradiation (Table 3) was very similar to that of 4 Gy x-rays. However, at day 7, the biological processes enriched at day 1 after neutron irradiation were not only retained, but additional DNA/RNA categories became significant, again only among down-regulated genes. tRNA metabolism (GO:0006399; *p* = 1.38E-06), RNA localization (GO:0006403; *p* = 1.52E-06) and transport (GO:0050658; *p* = 3.35E-06), and noncoding RNA metabolism (GO:0034660; *p* = 2.23E-05) were among the new biological processes over-represented at day 7.

**Table 2:** DNA and RNA metabolism-related biological processes in response to x-ray exposure

	Term	X4d1_UP	X4d1_DN	X4d7_UP	X4d7_DN
GO:0006259	DNA metabolic process	-	3.80E-10	-	-
GO:0016071	mRNA metabolic process	-	4.06E-10	-	-
GO:0006396	**RNA processing**	-	4.40E-10	-	-
GO:0006397	**mRNA processing**	-	5.67E-10	-	-
GO:0006412	translation	-	7.39E-10	-	-
GO:0006260	DNA replication	-	0.0003	-	-
GO:0010629	negative regulation of gene expression	-	0.0136	-	-
GO:0006261	**DNA-dependent DNA replication**	-	0.0181	-	-
GO:0006310	DNA recombination	-	0.0271	-	-
GO:0040029	**regulation of gene expression, epigenetic**	-	0.0382	-	-
GO:0042127	**regulation of cell proliferation**	-	0.0396	-	0.0097

Significantly enriched biological processes among genes downregulated in response to 1 and 4 Gy x-ray irradiation at days 1 and 7. Benjamini-corrected *p* values are shown. Processes unique to x-rays are in bold. X4d1: 4 Gy X-rays, day 1; X4d7: 4 Gy X-rays, day 7. UP, upregulated; DN: downregulated

**Table 3:** DNA and RNA-metabolism related biological processes in response to neutron exposure

	Term	N1d1_UP	N1d1_DN	N1d7_UP	N1d7_DN
GO:0006396	RNA processing	-	-	-	3.27E-18
GO:0050658	**RNA transport**	-	-	-	3.35E-06
GO:0000956	**nuclear-transcribed mRNA catabolic process**	-	-	-	0.0204
GO:0006260	DNA replication	-	9.59E-06	-	2.17E-06
GO:0006399	**tRNA metabolic process**	-	-	-	1.38E-06
GO:0006412	translation	-	0.0002	-	4.55E-11
GO:0008380	**RNA splicing**	-	0.0097	-	3.21E-13
GO:0034660	**ncRNA metabolic process**	-	-	-	2.23E-05
GO:0016071	mRNA metabolic process	-	0.0145	-	9.63E-14
GO:0006259	DNA metabolic process	-	2.06E-13	-	3.11E-11
GO:0006403	**RNA localization**	-	-	-	1.52E-06
GO:0045892	negative regulation of transcription, DNA-templated	-	0.0119	-	-
GO:0016444	**somatic cell DNA recombination**	-	0.0034	-	-
GO:0006401	**RNA catabolic process**	-	-	-	0.0427
GO:0016458	**gene silencing**	-	-	-	0.0358
GO:0006310	DNA recombination	-	0.0027	-	4.15E-05
GO:0051052	**regulation of DNA metabolic process**	-	-	-	0.0047

Significantly enriched biological processes among genes downregulated in response to 1 Gy neutron irradiation at days 1 and 7. Benjamini-corrected *p* values are shown. Processes unique to neutrons are in bold. N1d1: 1 Gy neutron, day 1; N1d7: 1 Gy neutron, day 7. UP, upregulated; DN: downregulated

Arguably, the most pronounced differences in the transcriptomic profiles of blood cells after neutron and x-rays were in the area of cellular metabolism. Neutron irradiation resulted in the downregulation of genes involved in metabolic processes, including coenzyme, hexose, and lipid biosynthetic processes. In contrast, exposure of mice to 1 Gy x-rays resulted in an overrepresentation of metabolic processes among up-regulated genes, especially those involved in lipid, cofactor, and vitamin biological processes (Table 4). These processes were detected at day 1, whereas they were no longer significant at day 7. Fatty acid metabolism (GO:0006631; *p* = 1.10E-42), lipid biosynthesis (GO:0008610; *p* = 5.83E-50), and sterol (GO:0016125; *p* = 4.44E-28) and steroid (GO:0008202; *p* = 1.54E-33) metabolism were among the top enriched biological processes.

**Table 4:** Lipid-related biological processes

	Term	Benjamini
GO:0008610	lipid biosynthetic process	5.83E-50
GO:0006631	fatty acid metabolic process	1.10E-42
GO:0008202	steroid metabolic process	1.54E-33
GO:0016125	sterol metabolic process	4.44E-28
GO:0016042	lipid catabolic process	3.98E-25
GO:0006869	lipid transport	1.70E-20
GO:0010876	lipid localization	7.57E-20
GO:0055114	oxidation-reduction process	1.02E-12
GO:0044242	cellular lipid catabolic process	1.27E-09
GO:0006662	glycerol ether metabolic process	0.0010
GO:0006638	neutral lipid metabolic process	0.0010
GO:0000038	very long-chain fatty acid metabolic process	0.0012
GO:0019216	regulation of lipid metabolic process	0.0027
GO:0055088	lipid homeostasis	0.0038
GO:0006775	fat-soluble vitamin metabolic process	0.0038
GO:0042157	lipoprotein metabolic process	0.0081
GO:0006637	acyl-CoA metabolic process	0.0105
GO:0034369	plasma lipoprotein particle remodeling	0.0118
GO:0006625	protein targeting to peroxisome	0.0118
GO:0043574	peroxisomal transport	0.0118
GO:0007031	peroxisome organization	0.0119
GO:0034754	cellular hormone metabolic process	0.0148
GO:0042632	cholesterol homeostasis	0.0202
GO:0006665	sphingolipid metabolic process	0.0267

Significant lipid biosynthesis processes among genes upregulated in response to 4 Gy x-rays at day 1 with Benjamini-corrected *p* values

DNA repair pathways were also differentially regulated, showing significant enrichment among down-regulated genes, especially after neutron irradiation. Thus, mismatch repair (MMR, mmu03430; *p* = 0.04) appeared downregulated 1 day after 1 Gy neutrons, whereas at day 7, besides MMR (*p* = 0.001), base excision repair (BER, mmu03410; *p* < 0.03), nucleotide excision repair (NER, mmu03420; *p* = 0.002), and non-homologous end joining (NHEJ, mmu03450; *p* = 0.02) were downregulated, as well. In contrast, no DNA repair pathways were significant after 1 Gy x-rays, whereas MMR (*p* = 0.03) and BER (*p* = 0.03) were downregulated after 4 Gy x-rays at days 1 and 7, respectively.

So far, DAVID analysis had suggested biological processes that appeared to be regulated in blood in response to radiation. These processes were either significant at only one time point, or if they persisted at both 1 and 7 days post-irradiation, they were consistently either up- or down-regulated. However, a careful examination of the biological processes revealed that the cell cycle regulation processes displayed a more complex temporal pattern. Specifically, cell cycle regulation processes were significant among down-regulated genes at day 1 after 4 Gy x-rays exposure. However the same processes were significant among up-regulated genes at day 7. At day 1, 54 cell cycle genes were differentially underexpressed, whereas, at day 7, 52 cell cycle genes were overexpressed. We searched for genes that appeared in both the downregulated and upregulated columns. We discovered 6 genes, namely *Mns1* (meiosis-specific nuclear structural protein 1), *Fzr1 (fizzy/cell division cycle 20 related 1 (Drosophila))*, *Ube2c* (ubiquitin-conjugating enzyme E2c), *Ccna2* (cyclin A2), *Anln* (anillin, actin binding protein)*, Smc2* (structural maintenance of chromosomes 2) and *Ncapd2* (non-SMC condensin I complex, subunit D2). We examined the expression of these genes in the samples that had been exposed to 1 Gy x-rays and 1 Gy neutrons and found 3 genes (*Fzr1*, *Ube2c*, *Ccna2*) with the same trend of temporal regulation after 1 Gy x-rays (*p* = 0.005) and after 1 Gy neutrons (*p* = 0.01).

**Fig. 4:**
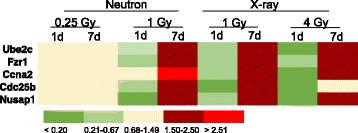
Heatmap illustrating relative expression of cell cycle genes bi-directionally regulated after neutron and x-ray irradiation. The mean (*n* = 6) fold change in gene expression relative to controls is color coded according to the scale bar at the bottom of the figure. Measurements were made by microarray analysis

Performing gene ontology analysis on the genes differentially expressed in samples exposed to 1 Gy neutrons using a less stringent *p* value (0.01), we discovered that, similar to the x-ray samples, cell cycle biological processes were downregulated at day 1 and upregulated at day 7. Sixty-six genes with cell cycle gene ontology annotations were underexpressed at day 1 and 44 genes were upregulated at day 7. Besides *Ube2c*, *Fzr1*, and *Ccna2* that were present in both the downregulated and upregulated lists, two more genes, *Cdc25b* (Cell division cycle 25b), and *Nusap1* (Nucleolar and spindle associated protein 1) showed a 0.24-fold (*Cdc25b*) and 0.16-fold (*Nusap1*) change at day 1, and 1.63-fold (*Cdc25b*) and 1.71-fold (*Nusap1*) at day 7 compared with controls. When these genes were examined against the x-ray gene profiles, they were found to be bi-directionally temporally regulated, as well. A heatmap of these five genes (*Ube2c*, *Fzr1*, *Ccna2*, *Cdc25b*, *Nusap1*) is depicted in Fig. 4. The levels of gene expression among control (unirradiated) samples were not significantly different between days 1 and 7.

### Quantitative real-time RT-PCR validation of cell cycle gene expression

**Fig. 5:**
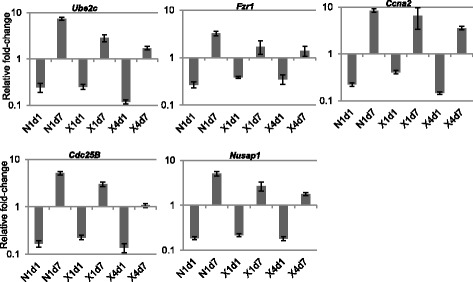
Gene expression measured by qRT-PCR. Expression of five genes (*Ube2c*, *Fzr1*, *Ccna2*, *Cdc25b*, and *Nusap1*) that were shown by microarray analysis to be bi-directionally regulated is depicted relative to controls and normalized to *Actb* expression. Data represent the mean ± S.E.M. (*n* = 6). N1d1: 1 Gy neutron, day 1; N1d7: 1 Gy neutron, day 7; X1d1: 1 Gy x-rays, day 1; X1d7: 1 Gy x-rays, day 7; X4d1: 4 Gy x-rays, day 1; X4d7: 4 Gy x-rays, day 7

We confirmed the expression pattern of the five cell cycle genes, shown by microarray analysis to be first under- then over-expressed after irradiation, by quantitative real-time PCR (Fig. 5). Analysis of gene expression of *Ube2c*, *Fzr1*, *Ccna2*, *Cdc25b*, and *Nusap1* by qRT-PCR confirmed that these genes are temporally regulated by irradiation and reverse the direction of their change during the first week after exposure. The fold-change of these genes in response to x-rays was in good agreement with the fold-change calculated by the microarray experiment, whereas qRT-PCR measurements indicated generally greater fold-changes than the microarrays in response to neutrons.

## Discussion

The overall goal of this study was to identify differentially regulated genes in response to neutron or x-ray irradiation and perform a comparative analysis of biological processes between the two types of radiation at time points spanning the range of interest for biodosimetry. Our data suggest that the gene transcriptional response varied widely depending on radiation quality, dose, and time since exposure. It should be noted that these characteristics of gene expression response may contribute to the apparently large number of “unique” genes responding to only one radiation quality. Previous studies have shown differences in the timing of gene expression responses at high and low doses, and following exposure to different radiation qualities. It is likely that many of the genes seen to respond to only one radiation quality in this study would show a response to the other radiation quality in a different time-dose combination. Some of the observed differences may also be attributable to the different nature of x-rays vs. neutrons. Up to 2/3 of damage from low-LET (e.g. x-rays) is due to indirect action, (mediated by free radicals), whereas high-LET neutrons cause direct damage to the DNA (Hall and Giaccia, 2012), which is more complex and difficult to repair, and may result in different signaling responses.

A small number of genes showed similar changes after exposure to neutrons and x-rays, and displayed a bidirectional mode of regulation. A similar temporal pattern of expression for some genes has been described previously in mice injected with ^137^CsCl [6]. In that study, genes were upregulated by day 2 or 3, and then downregulated by day 20 or 30 after isotope administration. Many gene ontology categories, including actin and the cytoskeleton and integrin signaling pathways, showed the same temporal pattern. In the present study, we identified a number of differentially expressed genes that were significantly different from controls at all radiation types and doses. A number of these genes were downregulated at day 1, and upregulated at day 7. Gene ontology analysis revealed that one biological function, cell cycle, was significant among down-regulated genes at day 1 and then significant among up-regulated genes at day 7. All other biological functions were either significant at one dose and time of irradiation, or they were uniformly up- or down-regulated irrespective of time.

Most of the genes differentially expressed in both neutron and x-ray exposures were related to immune response, and B and T cell physiology. These genes were downregulated starting at day 1 and reduced expression persisted until the end of the experiments at day 7. Widespread decreased expression of immune function genes has been shown previously in both human blood irradiated ex vivo, as well as in vivo mouse peripheral blood following ionizing radiation exposure or ^90^Sr as an internal emitter [22–24]. High-dose radiation (> 1 Gy) has been shown to disrupt immune cell functions, leading to increased cell death of blood cells in mice [25, 26]. Moreover, in patients with acute radiation syndrome, hematopoietic cell proliferation is inhibited by radiation exposure [27]. A preponderance of down-regulated genes has previously been associated with higher doses or later times after irradiation (e.g. [6]), perhaps reflecting greater damage or a failure of repair. In our study, even 0.25 Gy of neutron radiation resulted in the downregulation of genes involved in immune cell function.

A significant number of genes that were downregulated by both neutron and x-ray exposure at day 1 were related to DNA and mRNA metabolism, gene transcription, RNA processing and splicing. However, at day 7, these processes were no longer overrepresented following x-ray exposure, but they were present after neutron exposure. Moreover, in response to neutron exposure, these processes were enriched with additional related processes, such as tRNA metabolism/processing, RNA transport/localization, and noncoding RNA functions.

The contribution of DNA and RNA related functions in response to DNA damage has only recently been appreciated. A genome-wide siRNA screen looking for modulators of DNA damage signaling revealed that the largest number of hits were those targeting gene products responsible for nucleic acid metabolism, particularly those involved in mRNA binding and processing [28]. Furthermore, a phosphoproteomic analysis showed a close link between genome stability and RNA synthesis metabolism [29]. Likewise, it has been shown that the largest subset of ATM/ATR/DNA-PK substrates identified in a phosphoproteomic screen were proteins linked to RNA and DNA metabolism, particularly those proteins involved in posttranscriptional mRNA regulation [30]. These observations, employing different experimental approaches, highlight the importance of regulatory circuits controlling RNA metabolism and stability in DNA repair and checkpoint function. In addition to these findings, our study suggests that neutron (but not photon) irradiation affects biological processes enriched in tRNA regulation and RNA transportation and localization, as well as non-coding RNA metabolism and processing. tRNAs have been viewed as passive players involved in protein synthesis. However, recent evidence suggests that they have more active roles and tRNA modulation represents a mechanism by which cells achieve altered expression of specific transcripts and proteins. tRNA pools in cells can be divided into those that favor proliferation and those that support differentiation [31]. As a result, modifications in tRNA and their corresponding enzymes are implicated in diseases, including diabetes and cancer. For example, upregulation of certain tRNAs increases metastasis in breast cancer patients [32]. Furthermore, control of RNA transport and localization would be expected to impact on the rate of protein translation [33].

A difference observed between neutron and x-ray response was the enrichment in biological processes involved in lipid biosynthesis and metabolism that was seen only in response to x-ray exposure. It has long been known that the cellular targets of ionizing radiation, such as x-rays, are not limited to nuclear DNA, but that proteins and lipids in other cellular compartments, such as the plasma membrane [34], are also affected. The action of x-rays has been attributed to the generation of reactive oxygen species that oxidize DNA, lipids and proteins [35]. We can speculate that in response to x-rays, cells upregulate lipid, coenzyme, and vitamin biosynthetic and metabolic processes as a means of repairing the damage caused by x-ray irradiation to the cell membrane. The latter two processes could also serve as anti-oxidant responses [36]. In addition, fatty acid oxidation processes, which are also overrepresented in the x-ray irradiation, would be required by the cells to meet the energy demands of various metabolic processes.

In response to DNA damage, cells activate the DNA damage and repair signaling pathway. DNA damage that cannot be repaired efficiently leads to cell death or senescence. Although protein abundance and activity do not always follow gene expression changes, our Gene Ontology analysis suggests an apparent down regulation of several DNA repair pathways after neutron but not after x-ray irradiation, especially at day 7, perhaps reflecting a failure to repair the more complex damage resulting from high LET radiation. At day 1 after 1 Gy neutron exposure, GO analysis suggested suppression of MMR, whereas at day 7, in addition to MMR, NER, BER, and NHEJ genes were all significantly over-represented among downregulated genes. In contrast, exposure to 4 Gy of x-rays transiently downregulated expression of MMR genes at day 1 and BER genes at day 7. Homologous recombination, which along with NHEJ constitutes the major DNA double-strand break repair mechanism, was not significantly over-represented among differentially expressed genes. It has been shown previously that high-LET radiation induces complex DNA damage that is not easily repaired and NHEJ is not involved [37–40]. More recently, it has been shown that high-LET irradiation with protons or carbon ions causes a shift away from NHEJ toward HRR in the repair of double-strand breaks [41]. Consistent with these data, our observed downregulation of genes in the NHEJ and other DNA repair pathways in response to neutron exposure may reflect the fact that these lesions are not repaired by these processes. Regulation at the gene expression level suggests a potential mechanism for favoring the homologous recombination pathway in the attempted repair of neutron damage, and is worthy of further investigation.

A major biological function that is affected by radiation is the cell cycle. Cell cycle regulating genes are important determinants of radiosensitivity and cell fate in response to DNA damage. We studied the effect of neutron radiation on mouse cell cycle-regulated genes and compared it with that of x-rays. Unlike genes in other biological processes, which were either up or downregulated after neutron or x-ray irradiation, many cell cycle genes showed a bidirectional expression based on time. A group of 5 genes, namely *Ube2c*, *Fzr1*, *Ccna2*, *Cdc25b*, and *Nusap1,* were downregulated 1 day after irradiation, whereas the same genes were overexpressed 7 days post-irradiation. These temporal changes were further confirmed by qRT-PCR. A literature search revealed that these genes play important roles in the control of mitosis. Additionally, their protein products are related to the anaphase promoting complex/cyclosome (APC/C) either as subunits of APC/C (Ube2c, Fzr1) or as substrates (Ccna2, Nusap1).

The APC/C is an E3 ubiquitin ligase, which is composed of at least 14 core subunits. The APC/C is active during mitosis and G1 phase of the cell cycle. Because of its role in cell cycle regulation, APC/C is important for maintaining genomic integrity [42]. Furthermore, APC/C has been implicated in an array of diverse functions ranging from cell differentiation to apoptosis and senescence, as well as cellular metabolism, cell motility, and gene transcription through the degradation of specific substrates [42].

APC/C targets a large repertoire of substrates and recruits them for ubiquitylation via one of two co-activators, Cdc20 and Cdh1 (Fzr1, the mouse homolog) [42]. The physiological role of Cdh1 has been extensively studied in the context of human cancer, since downregulation of Cdh1 has been reported in many cancers, including those of prostate, ovary, liver, brain, and during the malignant progression of a B-lymphoma cell line. In mice, *Fzr1* heterozygosity results in the development of epithelial tumors, suggesting that Fzr1 may be a haploinsufficient tumor suppressor [43]. Downregulation of Cdh1 in post-mitotic neurons has been implicated in neurodegenerative diseases, such as Alzheimer’s disease [44].

In addition to its role in mitosis, Cdh1 has important functions in mediating DNA damage response to genotoxic stress [45] that ensure genomic integrity [46]. Cdh1-null cells fail to maintain DNA damage-induced G2 arrest and APC/C^cdh1^ is activated by x-irradiation-induced DNA damage (but not UV irradiation). Interestingly, the levels of mitotic cyclins in Cdh1^-/-^ cells after DNA damage were similar to those of wild-type cells. These data imply that cyclin A and cyclin B cannot be substrates for APC/C^cdh1^ when it is activated irregularly by DNA damage at G2 [47].

Protein ubiquitination-mediated degradation involves two distinct steps: the covalent attachment of ubiquitin to proteins catalyzed by the sequential actions of the activating (E1), conjugating (E2), and ligating (E3) enzymes, followed by the degradation of the poly-ubiquitylated protein by the 26S proteasome complex. For APC/C the E2 enzymes are Ube2c, which is one of the temporally controlled cell cycle genes in this study, and Ube2s [48], which is not differentially regulated in response to either neutrons or x-rays. Abundant experimental evidence has shown a role for Ube2c in human tumor initiation and progression. On the other hand, there are very few reports that implicate Ube2c in DNA damage response to radiation [49, 50]. Moreover, the mechanistic details of Ube2c response to radiation, as well as their pathophysiological significance remain unexplored.

APC/C regulates spindle formation by promoting the degradation of a number of spindle-binding proteins, including Nusap1 [51]. The nucleolar spindle-associated protein 1 (Nusap1) is a protein highly expressed in proliferating cells and interacts with microtubules [52]. Depletion of Nusap1 caused faulty mitotic spindles, aberrant chromosome segregation, and defective cytokinesis. Overexpression of Nusap1 caused microtubule bundling and cell cycle arrest at the G2/M checkpoint [53].

Cyclin A2 interacts with Cyclin-dependent kinase 2 and controls essential functions in DNA replication and cellular proliferation [54]. Cyclin A2 expression is associated with a poor prognosis in several types of cancer [55]. *Cyclin A2* mRNA as well as protein are cell cycle regulated [56] with mRNA and protein abundance increasing 4-fold and 20-fold, respectively, as cells progress from G1 to G2 phase. APC/C degrades Cyclin A2 at the end of mitosis, while mRNA persists longer than the protein in cells.

To fully appreciate the significance of the temporal differential expression of APC-related genes in the radiation response, we will require knowledge about the status of protein levels and their posttranslational modifications (e.g. phosphorylation). However, we can speculate that these changes may be relevant to cell cycle progression, and especially mitosis, thus ensuring genomic stability after irradiation.

Another cell cycle regulated gene that appears in the list of temporally bi-directionally expressed genes in the present study is *E2f2*. While this gene was consistently downregulated at day 1 post-irradiation, it always appeared upregulated at day 7. This was in sharp contrast with other E2f genes that showed no change at day 1 and downregulation (*E2f1*, *E2f3*, *E2f4*) at day 7 post neutron irradiation, or significant upregulation (*E2f1*, *E2f8*) at day 7 post x-ray irradiation. The E2f family of transcription factors has well known functions in the control of cell cycle, and E2f1-3, especially, in promoting G1/S cell cycle transition and thus cell proliferation [57]. Target genes of E2f include several hundred genes that are involved not only in DNA replication and cell cycle progression, but also in DNA damage repair, apoptosis, differentiation and development [58]. The role of E2fs in mitosis has been shown in cancer cells [59–63]. Although the specific function of E2f2 in response to radiation has not been studied, E2f2 transcript and protein levels increase in response to genotoxic stress and maintain genomic stability in neuronal cells [64]. Why *E2f2* shows bi-directional expression changes, whereas other E2f members (i.e., *E2f1*, *E2f3*, *E2f4*, and *E2f8*) do not, is currently not known.

## Conclusion

In the current work we identify genes that are differentially expressed following exposure to neutrons or x-rays, as well as genes that showed similar responses to the two radiation modalities. In summary, our results show that genes involved in cell cycle regulation are differentially regulated by neutron and x-ray radiation. However, a few cell cycle genes show a consistent temporal regulation that is common across the two radiation modalities. These genes are functionally interconnected and play important roles during mitosis.

We have found differing patterns of gene expression response to x-rays and neutrons that vary with both dose and time since exposure. These findings support the possibility of using gene expression to detect the neutron component of exposures resulting from an IND detonation, thus providing triage information more relevant to the actual radiation injury than an estimate of total dose alone. Further work will be needed to develop gene expression patterns specific to neutron exposure that would be useful for triage following an IND event. The identification of biomarkers predictive of both dose and type of radiation would be an important advancement in biodosimetry to determine an individual’s exposure and allow more accurate triage for further medical treatment.

The ultimate goal of our neutron studies is to investigate whether we can separately estimate the photon and the neutron component after a mixed photon/neutron exposure, and to develop gene expression signatures capable of discriminating between photon/neutron and pure photon exposures. Following our initial investigation of the gene expression response to neutrons, reported here, our future studies will focus on mixed exposures, including the possibility of synergistic responses.

## Additional files

Additional file 1: Excel file with 8 tabs containing a summary of all the differentially expressed genes by assay time and radiation type and dose. (XLSX 1628 kb)
Additional file 2: Excel file with complete output of gene ontology (GO) analyses summarized by dose. Results are in separate tabs for x-ray and neutron exposures. (XLSX 37 kb)

## Abbreviations

BER: Base excision repair
DAVID: Database for Annotation, Visualization, and Integrated Discovery
FDR: False discovery rate
GO: Gene ontology
IND: Improvised nuclear device
MMR: Mismatch repair
NER: Nucleotide excision repair
NHEJ: Non-homologous end joining
qRT-PCR: quantitative real-time Polymerase chain reaction
RBE: Relative biological effectiveness
